# Examining the themes of STD-related Internet searches to increase specificity of disease forecasting using Internet search terms

**DOI:** 10.1038/srep36503

**Published:** 2016-11-09

**Authors:** Amy K. Johnson, Tarek Mikati, Supriya D. Mehta

**Affiliations:** 1 University of Illinois at Chicago, School of Public Health, Chicago, 60608, USA; 2 Ann & Robert H. Lurie Children’s Hospital , Chicago, 60611, USA; 3 Chicago Department of Public Health, Chicago, 60604, USA

## Abstract

US surveillance of sexually transmitted diseases (STDs) is often delayed and incomplete which creates missed opportunities to identify and respond to trends in disease. Internet search engine data has the potential to be an efficient, economical and representative enhancement to the established surveillance system. Google Trends allows the download of de-identified search engine data, which has been used to demonstrate the positive and statistically significant association between STD-related search terms and STD rates. In this study, search engine user content was identified by surveying specific exposure groups of individuals (STD clinic patients and university students) aged 18–35. Participants were asked to list the terms they use to search for STD-related information. Google Correlate was used to validate search term content. On average STD clinic participant queries were longer compared to student queries. STD clinic participants were more likely to report using search terms that were related to symptomatology such as describing symptoms of STDs, while students were more likely to report searching for general information. These differences in search terms by subpopulation have implications for STD surveillance in populations at most risk for disease acquisition.

The Internet is an important source of health information, as it is anonymous, low- to-no cost, and can be accessed at any time. Millions of people search online for health- related information each day, most starting their search via a search engine such as Google^1^. Search terms can be downloaded and analyzed to detect patterns in relation to rates of disease to test the hypothesis that increases in specific search terms may be related to increases in rates of disease. Ginsberg *et al.* developed the Google-based application Google Flu Trends which can predict influenza 7–10 days earlier than traditional surveillance systems^2^. From this groundbreaking work published in 2009 came many subsequent studies. Following the popular Google Flu trends model, influenza has been successfully monitored via search engine data and outbreaks have been predicted in not only the United States but also in China, France and Spain^3^,^4^. In addition, multiple infectious diseases have been successfully predicted using Google Trends, such as dengue, West Nile Virus, tuberculosis, and more recently HIV^3^,^4^,^5^,^6^.

There are some limitations in using Internet search volume to forecast disease. First, accurate disease data is needed to train the initial predictive model. Search volume is not a stand-alone disease alert system, but rather an enhancement to established surveillance systems. Second, the algorithms of Internet search engines are adaptive to the individual user and present an unmeasured variable within the model^7^. Third, characteristics of Internet users who generate disease related searches as well as the content of their searches have not yet been examined. Finally, models have been shown to be sensitive to media hype (e.g., swine flu media coverage). Ideally, search terms used are reflective only of users with suspected or known disease, as opposed to Internet users in general. This is unlikely, and an increase in search volume could indicate a true finding or may indicate the need to further train the model by revising or weighting the search terms included. Investigators have proven that models with multiple search terms outperform models combining query volume into one variable, as those with multiple search terms allow for more nuanced discrimination^8^,^9^. For example, when Google Flu Trends erroneously predicted an outbreak of influenza it was based on the contribution of volume of a single search term, which once corrected, the model performed at its peak^10^.

In our preliminary study, we used publicly available Google Trends data to examine the association between sexually transmitted disease (STD) search terms and rates of disease by US state^11^. We found a positive and statistically significant correlation between rate of gonorrhea by state and volume of gonorrhea search terms^11^. Our follow-up study sought to characterize Internet users and STD-related search behavior. We found differences in user characteristics related to STD search prevalence, specifically that demographics were important predictors in a high-risk sample and sexual risk behavior was predictive in a low-risk sample. Here we present the analysis of reported Internet search terms to ascertain whether or not there are similarities or differences in the specific terms individuals use to access STD information. If there are differences in the content of the search terms, by demographics or risk behavior, specificity can be increased in the application of Google Trends for STD surveillance which enhances this modeling technique.

## Methods

Briefly, subjects were recruited from public STD clinics and from a university event. Recruitment was restricted to subjects aged 18–35 who were English-speaking. Survey completion took between 5 and 10 minutes and participants did not receive compensation. Data collection included demographic, sexual risk behavior and Internet search behavior. The Institutional Review Boards (IRBs) of the University of Illinois at Chicago and the Chicago Department of Public Health approved the study and study procedures were carried out in accordance with the approved protocol. Oral informed consent was obtained from all participants prior to survey completion; a waiver of written informed consent was obtained from both IRBs.

Data was collected via in-person interview and recorded verbatim by trained research assistants and the study PI. Open-ended responses were solicited by the question, “Please tell us what words or phrases you used to find the information.” All data was entered by the PI and later verified by a research assistant. Data files were imported into Dedoose, an online cloud-based mixed methods analysis program that specializes in allowing multiple users to code and display qualitative and quantitative data^12^. A strength of Dedoose is its ability to integrate mixed methods analysis, utilizing “descriptors” or quantitative data within the program.

Qualitative coding procedures followed grounded theory approach in which the codes applied reflect the content of the data rather than prior hypotheses^13^. The goal of using grounded theory approach was to highlight and explore similarities and differences within the data.

A multi-stage process of establishing inter-coder reliability and agreement was implemented^14^. The first rater read all response excerpts and developed a preliminary codebook consisting of 12 codes that emerged from the data. The second rater read all responses and then viewed the preliminary codebook. We used multiple raters and the process of multiple coding to enhance objectivity of analysis^15^. Multiple coders also allow for discussion and insight from establishing agreement. As the dataset was relatively small, the entire dataset was coded by both raters. After each rater reviewed the dataset the raters met to discuss adding, deleting or modifying codes (deleted 2 codes, added 2, modified 1); raters also discussed and documented their understating of each code. For example, the difference between applying the code “transmission” versus applying the code “prevention” was discussed. The process of concordance allows insights to the data which helps refine the coding frame^13^,^15^.

The Dedoose training module was used to randomly select 20 percent of all excerpts for both samples. Both raters coded these excerpts for training purposes and to establish a baseline reliability score prior to coding the entire dataset. In the majority of cases the disagreement involved omission of code and was agreed upon once reviewed. Including the passages where omission occurred the kappa coefficient was 0.81, representing a very good level of agreement; when the omissions were not included the kappa increased to 0.92, an excellent level of agreement reflecting very few disagreements in coding.

Both raters then coded the entire dataset for both samples. The dataset was compared for reliability, any divergence (n = 38) was discussed and reconciled, creating 100% inter-coder agreement. Divergence in general resulted from omission of codes, for example omitting a code in an excerpt that already had a code applied. After all divergences were reconciled the data was analyzed to detect themes and patterns by descriptors (e.g., response patterns in males compared to females). After all codes were analyzed, we selected only those with frequency of occurrence of at least 10% within each sample to compare between descriptors. However, we also present a summary of the codes that applied to a lower proportion of the sample to present the depth and breadth of the dataset fully.

Bivariate analysis was conducted using Pearson’s chi-square and Fisher’s exact test (when cell sizes contained fewer than 5 observations) to detect differences between the presence and absence of codes between samples, as well as the presence or absence of codes within samples by descriptors (e.g., age, sex). In effort to triangulate responses within the samples, we compared the presence or absence of each qualitative assigned code with the distribution of responses to the following statement: “I am going to read a list of reasons people use the Internet to search for information about STDs. In general, did you look for information because…” with the following yes/no categories: a) you wanted to learn more about STDs; b) you thought you might have an STD; c) you wanted to know how to prevent getting an STD; d) you wanted to know how to treat an STD; d) you wanted to find a place to get tested for STDs. Chi-square p-values are reported.

Google Correlate was used to identify correlated search terms in the United States with those search terms identified in the analysis. Google Correlate is a publicly available data tool that is a part of the Google Trends package. It enables users to enter search terms and to find queries with high correlations of the entered terms. Google Correlate is used to build multiple search query models in effort to refine and enhance the specificity of predictive models using Google Trends^16^.

## Results

Overall, 446 subjects were recruited from the public STD clinics and 279 students were recruited from the university. STD clinic patients were 57% male, median age 24, 30% non- Hispanic White, 51% non-Hispanic Black, and 19% Latino/Hispanic of any race. Students were 54% female, median age of 19, 32% Hispanic/Latino, 26% Non- Hispanic Black, 22% Non-Hispanic White, and 18% Asian.

Twelve codes emerged from the dataset in roughly two major content areas: seeking information about STDs using general terms (including prevention, testing and treatment) and accessing information based on symptoms (Table 1). One code “sex education” was only applied to the student excerpts. The term “education” occurred in 11% of the student excerpts and none of the clinic excerpts.

The clinic sample length of query ranged from 4 to 230 characters, with a median of 50 characters; the student sample ranged from 4 to 116 characters with a median of 41. The average English language query on Google is estimated at 20 characters or approximately 4–5 words^17^,^18^, thus our participants reported longer than average search queries. There were no demographic differences detected in the student sample in regard to length of query, however, in the clinic sample all respondents who reported queries over 150 characters were also Non-Hispanic Black.

**Figure 1:**
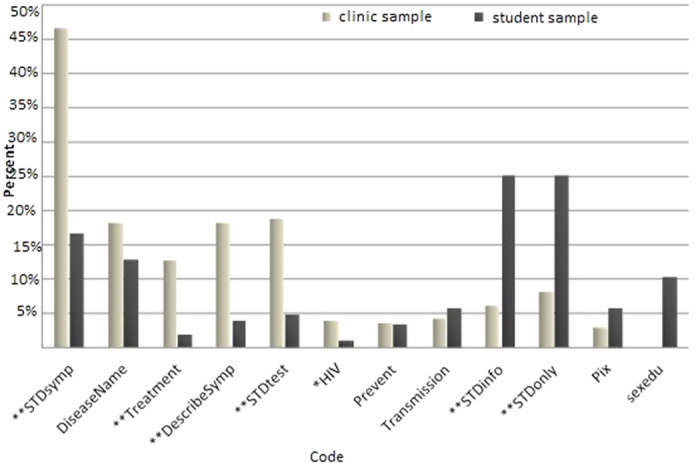
Frequency of qualitative code occurrence applied to STD-related search content in STD clinic (N = 446) and student (N = 279) samples. (*p < 0.05, **p < 0.01).

Nearly half (47%) of the clinic sample reported using the search term “STD Symptoms” compared to 17% of the student sample (p < 0.01; Fig. 1). In addition, clinic participants were significantly more likely than student participants to report describing STD-related symptoms, or searching using words related to treatment and testing. Student participants were significantly more likely than the clinic sample to report general terms, using “STD” as their only search term (26% vs. 8%; p < 0.01) or searching for general STD information (26% vs. 6%, p < 0.01). The clinic sample was more likely to report seeking information based on symptoms, reflected in codes for describing symptoms and using the phrase ‘‘STD symptoms’’, compared to the student sample, which was more likely to search for general information.

The top 5 codes that exceeded the 10% threshold are presented in Table 2 for the clinic participants and Table 3 for the student participants. The top codes for the clinic participants were codes that encompassed describing STD symptoms, using STD disease names, using the phrase “STD symptoms”, searching for STD testing information and/or searching for STD treatment. The top codes for the student participants were codes that encompassed searching for STD information, using the term “STD” as the only search term, using the phrase “STD symptoms”, searching for sexual education and/or searching using STD names. Two of the content areas using “STD symptoms” and searching using STD names were found in both the clinic and student samples among the top reported search queries.

In the clinic sample, females were more likely to report describing specific STD-related symptoms than were males (p = 0.04; Table 2). In addition, Non-Hispanic Blacks (22%) were more likely than both Non-Hispanic Whites (11%) and Hispanic/Latinos of any race (12%) to report searching for STD information by describing symptoms (p = 0.01). Those who were Hispanic/Latino were more likely to report using the search term STD symptoms compared to other races (p = 0.04). Those who were 25–29 years old were more likely to use the STD disease name in their search compared to any other age category (p = 0.01). Finally, those who used condoms were more likely than those who did not use condoms to report searching for STD test information (22% vs.14%, p = 0.02).

In the student sample, there were no differences in occurrence of the codes by race or sex (Table 3). Compared to other age groups, those who were older (25–29 years old) were more likely to report they searched using the term “STD Symptoms” (p = 0.01) or that they only used the term “STD” (p = 0.04). In terms of sexual behavior, men who have sex with women (MSW) were the most likely to report searching for STD information by typing “STD symptoms” (p = 0.04), there were no other differences in code occurrence by sexual behavior. Those who reported having a previous STD were more likely to search using a disease name (p = 0.05). Finally, those with zero sex partners in the past 6 months, were more likely to report searching for general STD information (p = 0.02).

### Results of Triangulation

Nine of the 11 codes applied to the clinic sample had no statistically significant relationship with the quantitative question and 9 of the 12 codes applied to the student sample had no statistically significant relationship. In the clinic sample, the code encompassing specific sexually transmitted disease name was associated with selecting “yes” to the response reflecting searching the Internet to learn more (p = 0.01). The codes reflecting searches related to transmission and prevention of STDs were associated with selecting “yes” to the response reflecting searching to learn how to treat an STD and how to prevent an STD (p = 0.01 and p = 0.03, respectively). In the student sample, the code for description of specific STD-related symptoms was related to selecting “yes” on the responses related to searching because you think you have a STD and for treatment information (Fisher’s exact p = 0.04; p = 0.02). The code for searches for STD test information was associated with selecting “yes” on the response related to searching to find a place to get tested (p = 0.02) and the code for searching for STD treatment was associated with selecting “yes” on the response related to searching to learn how to treat an STD (p = 0.01).

### Results of Google Correlate analysis

The direct term “STD symptoms” was reported by 47% of the clinic sample and 17% of the student sample. When entered into Google Correlate, the term “STD symptoms” generated many STD-related terms with high levels of correlation (0.87–0.94). The top 20 terms that were associated with “STD symptoms” are displayed in Table 4. Five of the twenty terms are not directly related to STDs (“how to talk to women”, “estrogen pills”, “pregnant symptoms” “talk to women” and “first trimester symptoms”). As disease name was often stated as a term used in searching for STD information (19% clinic; 13% student), “Chlamydia” was entered into Google Correlate. Sixteen of the top 20 search terms that were correlated with “STD symptoms” were also highly correlated with “Chlamydia.” Two of the terms that were generated by “STD symptoms” and not directly related to STDs (“how to talk to women”, “first trimester symptoms”) were not correlated with the search term “Chlamydia.” Search terms related to STD test, discharge (“thick discharge” “white discharge” “creamy discharge”), “STD” or STD treatment had few terms correlated that were related to STD information. For example, the term “STD” generated “gonorrhea” as the third highest correlated term at 0.924, however all of the other top 20 terms were unassociated with STDs. In addition, participant-generated search terms related to “sex education” and using the phrase “sexually transmitted infection” did not result in any Google correlated STD-related terms.

## Discussion

Using grounded theory approach, two main themes emerged: searching to find general STD information and searching based on symptoms. The themes emerged from the dataset, yet are intuitive based on our two samples. The assumption that a lower risk sample would search for more general information held true in our results, as did a higher risk sample searching based on symptoms of disease. There was an overlap in the highest frequency codes across samples of searching by disease name and using the phrase “STD symptoms”. In the student sample, risk behavior was associated with being more likely to have these codes applied, whereas in the clinic sample there were no associations found by demographic or risk behavior characteristics. This highlights that there is an overlap in the populations; although students as a whole are lower risk, at the individual level there is variation in exposure to STDs as well as search queries. This finding was previously undocumented, and aids in establishing specificity in disease forecasting.

We also noted that the query length median of our sample was double the reported average length of a Google query. This discrepancy may be due to data collection methods; participants were asked to report what they type to find STD information, they were not directly observed. Self-reported behaviors related to Internet search behavior may differ from actual behaviors, introducing measurement error. To our knowledge there has not been any published literature assessing the reported versus actual content of Internet searches. Google has an autocomplete feature which suggests the end of a search based on the first letters and words typed in. Thus, participants may have used more words to describe their search query then they would have if they were actually typing into Google. However, the qualitative codes did not account for query length and therefore our study’s main findings are not impacted by the data collection method. Additionally, we did not assess search process and patterns or type of device used to access information. All of these factors are not only important in understanding how users access STD-information online, but also in refining search terms used for predicting trends in disease.

Our efforts to triangulate the data were inconclusive, likely due to data collection methods and the multiple response format of the quantitative question. Participants were able to select multiple reasons why they searched for STD information and they were asked for their search terms generally. However, when associations were detected, they were between codes and responses that were congruent (i.e., code for STD test correlated with search reason “search for STD test information”). Future studies should consider a multi-stage approach to data collection in which qualitative responses are recorded and coded to inform the quantitative questions, which should then validate the coding scheme and content.

Google Correlate results produced 16 STD-related and strongly correlated search terms for two of the top reported terms (STD symptoms and disease name). The two terms were reported by both samples and validated using Google Correlate as a national sample. These terms could be used to build a multi-query predictive model for STD rates, by US state. Lampos *et al.* discovered a multi-query model enhanced the performance of the predictive capabilities of the Google Flu Trends model^9^. Accounting for variation within clusters of terms, weighting specific terms (to control for their overall contribution to the model), as well as supervising the machine learning (e.g., not completely automating the procedure) significantly improves inference^9^. The combination of search query data and disease data leads to better trained models for prediction^9^.

Study results should be interpreted in light of some limitations. All information was self-reported, including sexual risk behavior and previous STDdiagnosis. Surveys were confidential and anonymous, in effort to increase the validity of self-reported data.The study was cross-sectional and used convenience sampling, therefore, it is not representative of all Internet users or all persons with STDs. The study did target the population most affected by STDs who are also most likely to use the Internet (individuals ages 18–35). The primary weakness of previous studies analyzing the relationship between search trend data and disease rates is the absence of measuring and describing the content of disease-specific Internet searches. Our study addresses this gap and characterizes Internet users as well as the content of their STD-related searches. The mixed method approach allowed us to examine the content of queries from various angles leading to a more comprehensive understanding of search behavior. In addition, the combination of a high risk and lower risk sample allowed us to generate a rich heterogeneous qualitative data set^15^.

## Conclusion

This study described and analyzed search term query contents by content and sample characteristic. As models for disease monitoring are developed based on search term volume, it is important to understand if there are differences or similarities in search behavior and content in subpopulations that may be at risk for disease acquisition. Results from this study will help inform using Google Trends for STD surveillance by building queries specific to those most at risk of disease. Future studies should incorporate direct observation of search behavior and examine the influence of search patterns and Google’s autosuggest function, as well as testing predictive models using multiple correlated search queries.

## Additional Information

**How to cite this article**: Johnson, A. K. *et al.* Examining the themes of STI-related Internet searches to increase specificity of disease forecasting using Internet search terms. *Sci. Rep.* **6**, 36503; doi: 10.1038/srep36503 (2016).

**Publisher’s note**: Springer Nature remains neutral with regard to jurisdictional claims in published maps and institutional affiliations.

## Figures and Tables

**Table 1:** Search term codes and quotes from STD clinic and student sample.

*Code*	*Description*	*STD clinic patient quote*	*Student quote*
DescribeSymp	Description of STD Symptoms	“Abdominal pain, fishy odor, change in discharge”	“Itching down there”
DiseaseName	Use of STD by name	“Syphilis”	“Gonorrhea”
HIV	Mention of HIV or AIDS	“HIV and STD prevention” “HIV test clinic”	“HIV and STD”
Pix	Google images, searching for pictures of STDs	“How does STD look?”	“Google Images STD”
Prevent	Preventing STDs	“How safe are condoms”	“I want to know about STD before I have sex I look online”
STDinfo	General STD information	“What is STI mean”	“Health information for STD”
STDonly	Using the word STD, STI or Sexually Transmitted Infection and no other Terms	“STD”	“Sexually transmitted diseases”
STDsymp	Using the phrase STD symptoms or STI symptoms without a description of the Symptoms	“STD symptoms”	“Symptoms of STD”
STDtest	Looking for STD testing sites or information about testing procedures	“Free STD test”	“STD testing”
Treatment	Treatment or medication Information	“What can I do to treat chlamydia?”	“Treatment options for STD”
Transmission	How are STDs transmitted	“How do you spread STD?”	“STD risk- how do I get one?”
SexEdu*	Using the term education in Search	N/A	“Sex education online” “Free STD education”

*‘‘SexEdu” code only applied to student sample.

**Table 2:** Top qualitative codes by clinic sample characteristics.

	N	Describesymp	Diseasename	STDsymp	STDtest	Treatment
		n (%)	n (%)	n (%)	n (%)	n (%)
Sex						
Male	255	36 (14)*	44 (17)	108 (42)	46 (18)	28 (11)
Female	190	41 (22)	33 (17)	85 (45)	32 (17)	26 (14)
Race						
NH White	127	14 (11)^†^	14 (11)*	55 (43)*	29 (23)	13 (10)
NH Black	221	49 (22)	49 (22)	91 (41)	21 (10)	27 (12)
Hispanic/Latino, any race	82	10 (12)	12 (15)	40 (49)	24 (29)	12 (15)
Age						
18–19 years old	52	7 (13)	6 (11)^†^	26 (50)	11 (21)	6 (12)
20–24 years old	185	40 (22)	33 (18)	81 (44)	27 (15)	24 (13)
25–29 years old	138	22 (16)	30 (22)	59 (43)	30 (22)	11 (8)
30–35 years old	71	8 (11)	8 (11)	27 (38)	10 (14)	13 (18)
Sexual Behavior						
MSW	192	29 (15)	34 (18)	76 (40)	34 (18)	18 (9)
WSM	138	31 (22)	23 (17)	60 (43)	23 (17)	20 (14)
MSM	56	6 (11)	10 (18)	29 (52)	9 (16)	10 (18)
WSWM	49	10 (20)	10 (20)	24 (49)	8 (16)	6 (12)
Previous STD						
Yes	222	42 (19)	46 (21)	99 (45)*	32 (14)*	29 (13)
No	166	35 (16)	31 (14)	91 (42)	42 (20)	25 (12)
Number of sex partners						
1 partners	117	27 (23)	22 (19)	53 (45)	20 (17)	21 (18)
2–3 partners	177	30 (17)	30 (17)	74 (42)	34 (19)	18 (10)
4 or more partners	142	20 (14)	25 (18)	64 (45)	22 (15)	15 (11)
Condom use						
Used condom	165	25 (15)	33 (20)	71 (43)	37 (22)*	20 (12)
Did not use condom	273	52 (19)	44 (16)	119 (44)	38 (14)	34 (12)

MSW = Men who have sex with Women; WSM = women who have sex with men; MSM = men who have sex with men; WSWM = women who have sex with women and men.

*p < 0.05; ^†^p < 0.01.

**Table 3:** Top qualitative codes by student sample characteristics.

	N	STDinfo	STDonly	STDsymp	SexEdu	diseasename
		n (%)	n (%)	n (%)	n (%)	n (%)
Sex						
Male	126	26 (21)	22 (17)	20 (16)	10 (8)	9 (7)
Female	153	23 (15)	29 (19)	14 (9)	16 (10)	16 (10)
Race						
NH White	64	12 (19)	8 (13)	8 (13)	4 (6)	6 (9)
NH Black	73	13 (18)	14 (19)	9 (12)	8 (11)	9 (12)
Hispanic/Latino, any race	91	15 (16)	18 (20)	13 (14)	6 (7)	6 (7)
Asian	51	10 (20)	11 (22)	4 (8)	3 (6)	5 (10)
Age						
18–19 years old	144	26 (18)	29 (20)*	18 (13)*	11 (8)	12 (8)
20–24 years old	185	24 (13)	20 (11)	13 (7)	10 (5)	14 (8)
25–29 years old	8	1 (12)	2 (25)	3 (37)	0	0
Sexual Behavior						
MSW	86	19 (22)	16 (19)	17 (20)*	5 (6)	4 (5)
WSM	81	13 (16)	17 (21)	7 (9)	9 (11)	9 (11)
MSM	15	5 (33)	4 (27)	1 (7)	2 (13)	3 (20)
WSWM	17	6 (35)	4 (24)	0	2 (12)	1 (6)
Previous STD						
Yes	34	6 (18)	4 (12)	3 (9)	5 (15)	6 (18)*
No	166	37 (22)	37 (22)	22 (13)	13 (8)	12 (7)
Number of sex partners						
0 partners	21	5 (24)*	4 (19)	3 (14)	0	2 (9)
1 partner	94	19 (20)	24 (26)	7 (7)	12 (13)	8 (9)
2 partners	46	3 (6)	12 (26)	10 (22)	3 (7)	2 (4)
3 or more partners	43	2 (5)	9 (21)	4 (9)	4(9)	7 (16)
Condom use						
Used condoms	126	27 (21)	29 (23)	16 (13)	10 (56)	10 (8)
Did not use condoms	74	18 (24)	13 (18)	9 (12)	8 (44)	7 (9)

MSW = Men who have sex with Women; WSM = women who have sex with men; MSM = men who have sex with men; WSWM = women who have sex with women and men.

*p < 0.05.

**Table 4:** Google Correlate results for the United States: Top reported search terms generated by STD clinic and student samples.

	*STD Symptoms*	*Chlamydia*	*STD test*	*Thick discharge*	*STD treatment*	*STD*
Chlamydia	0.943	—	—	—	—	—
STD symptoms in men	0.935	0.919	—	—	—	—
Chlamydia Treatment	0.906	0.918	—	—	0.815	
Chlamydia symptoms	0.903	0.900	—	—	—	—
Chlamydia in men	0.902	0.913	—	—	—	—
*How to talk to Wome*n	0.900	—	—	—	—	—
Signs of STD	0.898	0.929	—	0.944	—	—
STD symptoms in women	0.893	—	—	—	—	—
Gonorrhea Symptoms	0.889	0.984	0.854	—	0.807	0.924
*Estrogen pills*	0.888	0.922	—	0.931	—	—
*Pregnant Symptoms*	0.882	0.925	—	0.965	—	—
*Talk to women*	0.881	0.902	—	—	—	—
Cure Chlamydia	0.879	0.932	—	—	—	—
Treat Chlamydia	0.878	0.938	—	—	—	—
Syphilis symptoms	0.878	—	—	—	—	—
Discharge	0.877	0.940	—	—	—	—
Thick Discharge	0.875	0.917	—	—	—	—
Gonorrhea	0.872	0.941	—	—	—	—
*First trimester Symptoms*	0.871	—	—	—	—	—
Milky white discharge	0.871	0.905	—	0.955	—	—

*Terms in italics not directly related to STDs.*

Table reports Pearson correlation coefficient.

*top codes- sex education, sexual health education, sexually transmitted infection, and STD did not produce any STD-related search terms.
